# Associations between postrace atrial fibrillation and measures of performance, racing history and airway disease in horses

**DOI:** 10.1111/jvim.16878

**Published:** 2023-09-23

**Authors:** Laura C. Nath, Adrian Elliott, Andre La Gerche, Joe Weir, Grace Forbes, Gijo Thomas, Samantha Franklin

**Affiliations:** ^1^ University of Adelaide Roseworthy Australia; ^2^ University of Adelaide Adelaide Australia; ^3^ Baker Heart and Diabetes Institute Melbourne Australia; ^4^ Hong Kong Jockey Club Hong Kong; ^5^ Racing Victoria Melbourne Australia

**Keywords:** Australia, EIPH, epidemiology, equine, exercise induced pulmonary hemorrhage, exercise volume, heart, Hong Kong, poor performance, regulation, retrospective, rhythm, risk factors

## Abstract

**Background:**

Atrial fibrillation (AF) is the most common performance limiting arrhythmia in racehorses. High dose exercise and airway disease promote AF in humans. Few studies have investigated epidemiological factors associated with AF in horses.

**Objectives:**

Explore variables relating to performance, exercise volume and postrace endoscopic findings in horses with AF.

**Animals:**

A total of 164 horses with poor race performance and postrace AF were compared to 321 horses with poor performance without AF (PP) and 314 horses performing to expectation (TE).

**Methods:**

Horse‐level and race‐level variables for horses racing in Australia and Hong Kong from 2009 to 2021 were compared using univariable and multivariable logistic regression. Postrace endoscopic exercise‐induced pulmonary hemorrhage (EIPH) and tracheal mucus accumulation (TMA) grades for AF and PP horses were compared using chi‐squared analysis.

**Results:**

Variables that were significant in the multivariable model of AF compared to TE were distance (lengths) behind the winner, (odds ratio [OR]; 95% confidence interval [95% CI], 1.41 [1.32‐1.51], *P* < .0001), cumulative prize money per start before the event (OR [95% CI] 1.02 [1.01‐1.03], *P* = .01) and age (OR [95% CI] 0.72 [0.55‐0.92], *P* = .01). More AF horses had EIPH grade ≥3 (23/109; 21.1%) than PP horses (7/213; 3.3%; OR [95%CI] 7.9 [3.3‐20.2], *P* < .0001).

**Conclusions and Clinical Importance:**

Acute race performance was substantially impaired by AF but career earnings before the event were not inferior. Exercise volume did not promote AF. Higher grades of EIPH found in AF horses suggests a mechanistic relationship between these conditions.

**Abbreviations**
AF: atrial fibrillation
EIPH: exercise‐induced pulmonary hemorrhage
PAF: paroxysmal atrial fibrillation
PP: poorly‐performing horse without cardiac arrhythmia
TE: horse performing to expectation
TMA: tracheal mucus accumulation

## 1 INTRODUCTION

Atrial fibrillation (AF) impairs performance^1^ and has an estimated prevalence among racehorses of 0.11% to 4.9%.^2^, ^3^, ^4^, ^5^ In racing horses, AF typically is paroxysmal^3^, ^4^, ^5^, ^6^, ^7^ and can be challenging to identify and diagnose in the absence of continuous ECG recordings or rapidly applied devices.^2^, ^8^, ^9^, ^10^ Implantable ECG loop recorders have documented paroxysmal atrial fibrillation (PAF) in a high proportion of poorly‐performing standardbred racehorses.^9^ Recurrence of AF is common,^5^, ^11^, ^12^, ^13^, ^14^ and progression from paroxysmal to persistent AF is suspected.^14^ These findings suggest that the AF burden (ie, the combined frequency and duration of episodes) in racehorses could be higher than is currently recognized and that short, paroxysmal episodes might go undetected but could still limit performance.

In human athletes, electrical, structural and functional cardiac remodeling occurs in association with strenuous exercise.^15^ Chamber enlargement along with enhanced parasympathetic activity, as indicated by sinus bradycardia, are well recognized cardiovascular adaptations.^15^, ^16^, ^17^ Exercise volume is defined as the frequency, duration and intensity of strenuous physical activity.^18^ High exercise volumes are thought to promote AF development.^16^, ^17^, ^18^, ^19^, ^20^, ^21^ In a similar manner to humans, horses undergo cardiac adaptations to exercise including chamber enlargement,^22^, ^23^ electrical remodeling and increased parasympathetic activity.^24^, ^25^ Thoroughbred and standardbred horses have the highest rates of AF and it most often occurs when these horses are young and in race competition.^26^ Older cohorts with AF, including former racehorses that have transitioned to equestrian disciplines, are more likely to have clinically identifiable structural cardiac pathology.^26^ In thoroughbred racehorses in the United Kingdom, the rates of circulatory system incidents increased with age.^4^ In Japanese thoroughbreds, it was found that older age and turf racing were associated with incident AF.^3^ In standardbred racehorses, 1 study found age to be a risk factor for AF development,^2^ whereas another study found no association between age and frequency of cardiac arrhythmias.^27^ Based on these studies, high volumes of strenuous exercise might promote AF in horses.

The impact of upper and lower airway disease on the development of cardiac arrhythmias including AF in horses is unclear. In humans, sleep‐disordered breathing contributes to recurrent episodes of hypoxemia and hypercapnia along with marked fluctuations in autonomic tone.^28^, ^29^ These conditions are thought to contribute to AF development, particularly in young people without structural heart disease.^28^, ^29^ Decreased lung function associated with chronic obstructive pulmonary disease is also a risk factor for AF in humans.^30^ Hypercapnia and hyperlactatemia have been identified in horses with exercise‐associated cardiac arrhythmias.^31^ However, conflicting results are reported regarding the relationship between cardiac arrhythmias and upper and lower airway disease, with 1 study finding that upper airway abnormalities were associated with arrhythmias^32^ and another study finding no association with arrhythmias and either upper or lower airway disease.^31^ In horses presented to the hospital with AF, exercise intolerance was the most commonly reported sign, followed by dyspnea and epistaxis.^27^ However, an association between epistaxis, presumably caused by severe exercise‐induced pulmonary hemorrhage (EIPH), and AF has not been clearly shown.^3^ Endoscopy is a sensitive test for EIPH and tracheal mucus accumulation (TMA) and can aid in the diagnosis of lower airway disease.^33^, ^34^, ^35^, ^36^, ^37^, ^38^

Our objectives were to explore associations between AF and potentially relevant risk factor variables relating to performance, exercise volume and postrace endoscopic findings. Our hypotheses were: (1) That horses with postrace AF would have poor performance before the diagnosed event, possibly because of undetected PAF; (2) Horses with AF would have higher volumes of exercise before the diagnosed event; and, (3) Horses with AF would have evidence of lower airway disease.

## 2 MATERIALS AND METHODS

### 2.1 Study design

A retrospective longitudinal cohort study of horses racing in Hong Kong from 15th February 2009 to 29th February 2017 and in Victoria, Australia from 17th February 2018 to 16th August 2021 was performed. The study complied with the Australian code for the care and use of animals for scientific purposes. The information retrieved for this study was collected as part of routine race day protocols and no additional procedures were performed on the horses.

### 2.2 Case selection, case definition, and inclusion criteria

#### 2.2.1 Selection of case horses

Industry records were used to identify all horses with postrace heart rhythm abnormalities during the previously defined period. Cases were horses that were examined postrace by an on‐course veterinarian for below expectation performance and were inspected in Section 2.3 below. The on‐course veterinarian identified a cardiac arrhythmia by auscultation and, according to race‐day protocols, an ECG was acquired within 60 minutes. Electronic PDF copies of ECGs performed on course at the time of the event were reviewed by 2 experienced veterinarians (L.N. and S.F.) to confirm the diagnosis of AF.

#### 2.2.2 Selection of comparison horses

Comparison horses competed at the same race meeting and (i) were inspected postrace for poor performance (PP) but were not diagnosed with a cardiac arrhythmia or (ii) performed to expectation (TE) and were not inspected. Four horses for comparison were selected for each AF horse, 2 from the PP group and 2 from the TE group. Horses in the PP group underwent postrace inspection as described in Section 2.3 below. An ECG was not performed on horses in the PP group. Horses that performed to expectation did not undergo postrace inspection. Comparison horses were selected using the following method: For each AF event, all horses that were inspected postrace at the same meeting were identified and entered into a spreadsheet. They then were sorted by the RANDOM number function and the first 2 horses were selected. This same method was used for selection of horses that performed to expectation. The race records in the online database for Hong Kong (HK)^39^ and Victorian^40^ horses were reviewed. Any horses that had previous episodes of cardiac arrhythmia identified in the records at any time in their careers were excluded from the study.

#### 2.2.3 Removing duplicates

Horses with AF and comparison horses that were selected according to the above criteria were entered into a spreadsheet. Horses that appeared in the spreadsheet on more than 1 occasion were identified. Duplicates were sorted by the RANDOM function and the second listed duplicate was excluded from the study. This method facilitated the comparison of exercise volume between AF horses and comparison horses because any listed event was equally likely to be selected.

### 2.3 Postrace veterinary inspection, ECG, and endoscopy

Protocols for postrace veterinary inspection in HK^5^, ^41^ and Victoria^8^, ^42^ have been described previously. Briefly, horses were subject to veterinary examination mainly at the request of race‐day stipendiary stewards, when horses delivered a disappointing race performance, an abnormality was noted during or after the race, or based on the jockey's postrace report. Further detail of postrace inspections is provided in the Supplementary Material. Veterinary inspections were performed within 10‐60 minutes of the race and consisted of cardiac and respiratory auscultation, lameness examination and palpation of musculoskeletal structures, and video tracheobronchoscopic examination. Protocol dictates that all of these procedures are attempted routinely. Tracheobronchoscopic examination was not performed in horses that were difficult to handle. Horses with normal cardiac auscultation findings did not have an ECG performed. In horses that had a cardiac rhythm abnormality identified by auscultation, ECG was performed within 60 minutes of race completion with a base‐apex configuration using either the Televet 100 or Schiller MS‐3 ECG V 2.05 ECG units (Schiller, Baar, Switzerland) in HK or a smartphone ECG, AliveCor Veterinary ECG monitor or Kardia 6‐lead ECG (Alive Technologies Pty Ltd, Ashmore, Australia) in Victoria.

### 2.4 Data analysis

#### 2.4.1 Software

Statistical analyses were performed using Graphpad Prism version 9^a^ (GraphPad Software, San Diego, USA). Statistical significance was set as *P* < .05.

#### 2.4.2 Risk factors associated with AF

Risk factor variables were selected to provide information about exercise volume and performance. Risk factors related to performance were: rating, prize money and distance behind the winner. Risk factors related to exercise volume were: race distance, weight carried, age, cumulative number of starts and cumulative race distance. Risk factors related to lower airway disease were: EIPH and TMA grade. Rating, provided as a numerical value, was determined prerace by the stipendiary handicapper. The rating given takes into account the overall performance of the horse with strong emphasis on most recent form. A higher rating reflects better performance. Cumulative prize money, cumulative number of starts and cumulative race distance were calculated by summing all race earnings, all starts and all race distances respectively, before the AF episode (or equivalent start for TE or PP horses).

Industry databases were used to extract historical data (age and race history) for each horse. On the race day of the AF episode (or equivalent start for PP or TE horses), rating, race distance (meters, converted to km for logistic regression), weight carried (kg) and distance in lengths behind the winner were examined as continuous variables. Historical data leading up to the event were: age (years), cumulative number of starts, cumulative race distance and cumulative prize money, and each of these were examined as continuous variables. Age at the time of the race meeting was determined for each horse by calculating the number of days between birth and the race meeting date, measured as a continuous variable and divided by 365. Before analysis, prize money in HK dollars was divided by 7.85 and in Australian dollars was divided by 1.45 to convert to US dollars, then divided by 1000 and reported as per 1000 US dollars. Interval data were retrieved from the industry race records and investigated as follows: the summed number of starts, race distance and prize money in the 30 days before and including the event were recorded as well as in the 30‐60, 60‐90 and 90‐180 days prior and examined as continuous variables. The race distance per start, and prize money per start, were calculated by dividing the prize money or race distance, respectively, during the interval by the number of starts during the interval. The interval race distance per start and prize money per start for each horse were examined as continuous variables.

#### 2.4.3 Analysis of postrace endoscopic findings

Postrace endoscopic examination was performed as part of the postrace inspection in AF horses and PP horses. Exercise‐induced pulmonary hemorrhage grade^33^ and TMA grade^35^ were determined by the attending veterinarian and recorded. In addition, EIPH grade ≥3 and TMA grade ≥2 were considered clinically important.

#### 2.4.4 Statistical methods

The summary statistics are presented as frequencies and percentages, and continuous variables as median (interquartile range [IQR]). The data were checked for normality using the Shapiro‐Wilk test.

Exploration of the risk factors associated with AF for PP and TE horses initially was assessed using 1‐way analysis of variance (ANOVA) and the Kruskal‐Wallis test with Dunn's correction for multiple comparisons. This analysis was performed before logistic regression to identify differences between AF horses, PP and TE groups and to identify any important differences between the 2 control groups.

Univariable conditional logistic regression was performed to obtain odds ratios (OR) with 95% confidence intervals (CI) and evaluate associations between all race day and historical variables of interest and binary outcomes (AF or no AF). Missing data were left as missing.

The binary outcomes examined were as follows:

Model 1: Comparison to PP horses: To account for selection of horses for postrace inspection by stipendiary stewards as a potential source of bias, horses with AF were compared to a group of horses that also had been selected for inspection but found not to have an arrhythmia.
Model 2: Comparison to TE horses: This analysis was performed to evaluate differences between AF horses with poor performance and normally‐performing horses.

The multivariable logistic regression models were built using a manual backwards stepwise method of elimination based on the likelihood ratio test *P* value. Spearman correlation coefficients were calculated for age in comparison to cumulative starts, cumulative distance, and cumulative prize money. This analysis identified that these variables were correlated with age and therefore were excluded from the multivariable model. Interval data variables were not submitted to the multivariable model because these events were contained within the cumulative prize money per start and cumulative distance per start reported in the historical data, which were submitted to the multivariable model. Interactions among other variables in the model was assessed by visual inspection of the correlation matrix. Race distance was removed at this step because this variable was correlated with distance behind the winner. The jurisdiction (Australia vs HK) was forced into all models to control for variation between sites. Goodness‐of‐fit was assessed using a combination of the Hosmer and Lemeshow test and the log‐likelihood ratio (G squared test) test. Variables were retained if their exclusion from the model impaired the goodness‐of‐fit.

For horses that had postrace respiratory endoscopy, the outcomes of interest were as follows: EIPH grade > 0, EIPH grade ≥3, TMA grade > 0, and TMA ≥2. The association between AF and EIPH and TMA was assessed using chi‐squared and Fisher's exact contingency table analysis.

## 3 RESULTS

### 3.1 Animals

**FIGURE 1:**
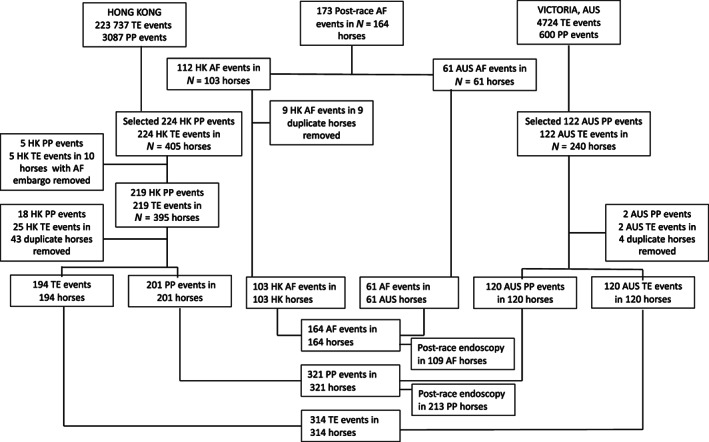
Flow chart demonstrating recruitment of horses for the study.

A flow chart demonstrating the selection of horses for inclusion in the study is provided in Figure 1. Atrial fibrillation was confirmed on 173 occasions in 164 horses during the study period. Of these, 112 events occurred in HK and 61 in Victoria. Comparison data were included for 321 PP horses and 314 TE horses after removal of duplicates and exclusion of horses in the PP or TE groups if they had a historical episode of cardiac arrhythmia in their racing record.

### 3.2 Risk factors associated with AF

#### 3.2.1 Univariable analysis

**FIGURE 2:**
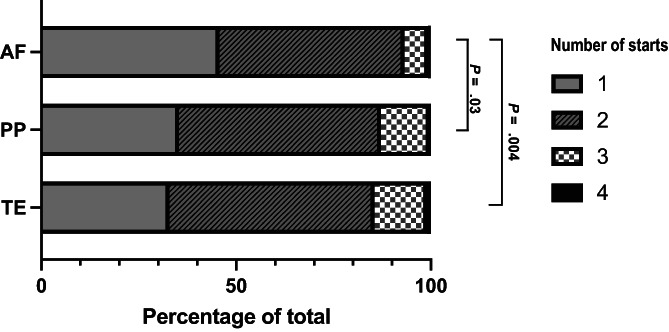
Stacked bar chart showing the number of starts in the 0‐30 days before and including the diagnosed AF episode or control start for PP and TE horses. Data show the number of horses having 1, 2, 3, or 4 starts in this interval expressed as a percentage of the total number of horses in the group. Starts were nonnormally distributed and ranked starts were compared Kruskal‐Wallis test with Dunn's correction for multiple comparisons. Significant differences between groups are marked with black lines and *P* values reported in the figure.

**FIGURE 3:**
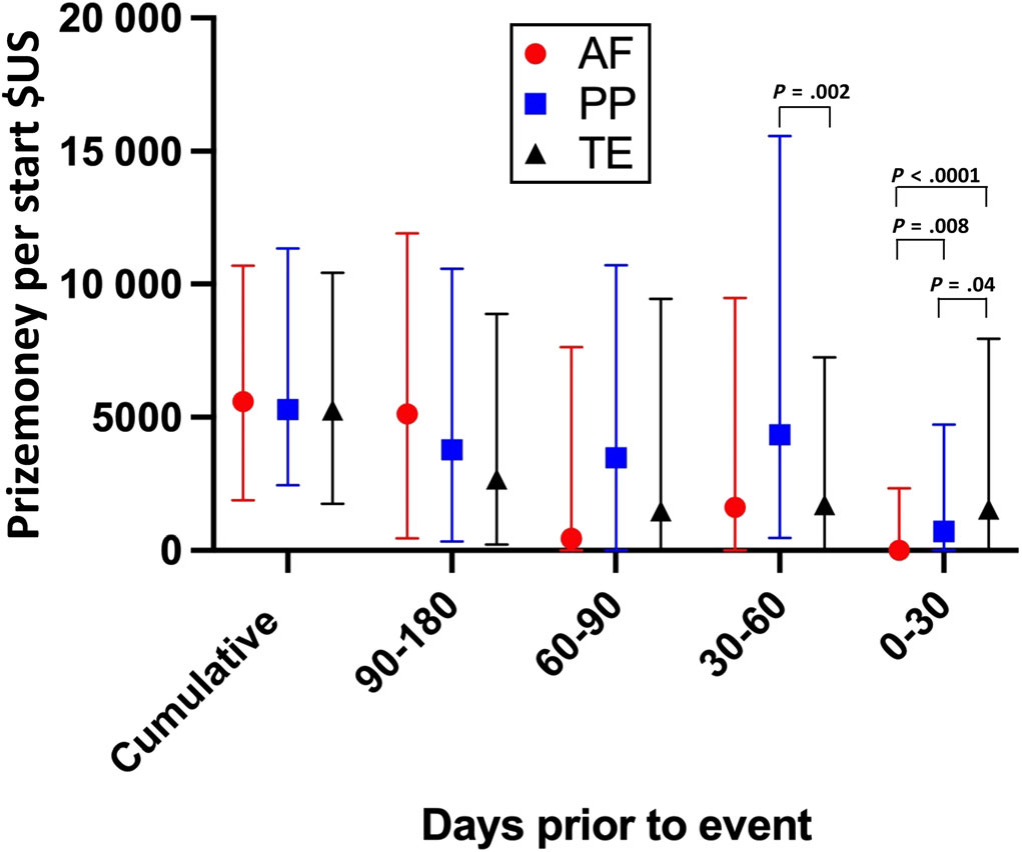
Comparison between cumulative prize money per start and prize money per start in the intervals 0‐30, 30‐60, 60‐90, and 90‐180 days before the event for AF, PP, and TE horses. Central bar represents the median and error bars represent the interquartile range. Kruskal‐Wallis test with Dunn's correction for multiple comparisons, significant differences between groups are marked with black lines and *P* values reported in the figure.

The results of the Kruskal‐Wallis test with multiple comparisons for each of the 30 examined variables are provided in Table S1. Results for AF horses were different from the PP and TE horses for the following investigated variables: Horses with AF were distanced (measured in lengths) further behind the winner, median (IQR), 20.93 (13.56‐29.88) compared to PP (7 [4.8‐9.8], *P* < .001) and TE (4.3 [2‐6.8], *P* < .0001). Horses with AF had fewer ranked starts in the 0‐30 days before and including the event (median [IQR], 2 [1, 2]), compared to PP (2 [1, 2], *P* = .03) and TE (2 [1, 2], *P* = .004; Figure 2). Horses with AF had less prize money (US $) in the 0‐30 days before and including the event (median [IQR], 0 [0‐5371]) compared to PP (1428 [0‐9439], *P* = .007) and TE (2831 [0‐14 666], *P* < .0001). Horses with AF had less prize money (US $) in the 30‐60 days before the event (median [IQR], 0 [0‐4784]) than PP horses (455 [0‐10 877], *P* = .01). Horses in the TE group also had less prize money (US $) in the 30‐60 days before the event (median [IQR], 0 [0‐4459], *P* = .003) than PP horses. A chart depicting prize money per start at each of the studied intervals is provided in Figure 3. Horses with AF had less prize money per start in the 0‐30 days before and including the event (median [IQR], 0 [0‐2324]) compared to PP horses (703 [0‐4720], *P* = .01) and TE horses (1540 [0‐7955], *P* < .0001). Horses in the PP group had less prize money (US $) in the 0‐30 days before and including the event than TE horses (*P* = .04). Horses in the TE group had less prize money per start in the 30‐60 days before the event (median [IQR], 0 [0‐4459], *P* = .003) than PP horses. No significant differences were found in cumulative starts, cumulative distance, cumulative prize money, distance per start or prize money per start at any other time interval.

Results of univariable logistic regression evaluating race day and historical data are provided in Table S2. Results for AF horses were different from PP and TE horses for the following investigated variables: Horses with AF were distanced further behind the winner (lengths), than PP horses (OR [95% CI], 1.17 [1.13‐1.21], *P* < .001) and TE horses (OR [95% CI], 1.32 [1.26‐1.40], *P* < .0001). Horses with AF had higher cumulative distance (km) per start, (median [IQR], 1.4 [1.2‐1.7]) compared to PP horses (1.4 [1.2‐1.6], OR [95% CI], 1.55 [1.05‐2.48], *P* = .02), but not compared to TE horses. Horses with AF had higher cumulative prize money (US $1000) per start (median [IQR], 5.6 [1.8‐10.7]) compared to PP horses (5.3 [2.4‐11.4]; OR [95% CI], 1.01 [1.00‐1.02], *P* = .05), but not compared to TE horses.

#### 3.2.2 Multivariable analysis

Results of the multivariable logistic regression models are presented in Table 1. In model 1, variables that were retained in the multivariable model of AF compared to PP were distance (lengths) behind the winner (OR [95% CI], 1.19 [1.15‐1.24], *P* < .001), cumulative prize money per start before the event (OR [95% CI] 1.02 [1.01‐1.04], *P* = .006) and age (years; OR [95% CI] 0.76 [0.61‐0.93], *P* = .01). In model 2, the same variables were retained in the multivariable model of AF compared to TE, distance (lengths) behind the winner, (OR [95% CI], 1.36 [1.29‐1.45], *P* < .0001), cumulative prize money per start before the event (OR [95% CI] 1.02 [1.01‐1.03], *P* = .01) and age (years; OR [95% CI] 0.72 [0.55‐0.92], *P* = .01).

**TABLE 1:** Findings from multivariable logistic regression.

Model 1: Horses with Atrial fibrillation (AF, N = 164) are compared to other poorly performing horses (PP, N = 321).				
Variable	Median (IQR)	OR	OR 95% CI	*P* value
Intercept		0.11	0.04‐0.30	<.0001
Jurisdiction (HK vs AUS)		1.68	0.97‐3.0	.07
Distance behind winner (lengths)				
AF	20.93 (13.56‐29.88)	1.19	1.15‐1.24	<.0001
PP	7 (4.75‐9.75)			
Cumulative prizemoney (US $1000)/start				
AF	5.6 (1.8‐10.7)	1.02	1.01‐1.04	.004
PP	5.3 (2.4‐11.4)			
Age				
AF	4.8 (4.1‐6.1)	0.76	0.61‐0.93	.01
PP	5.0 (4.1‐6.0)			
Comparison with PP. Tjur's r^2^ = 0.42, area under ROC curve (95% CI) = 0.88 (0.85‐0.92), *P* < .0001. Hosmer‐Lemeshow statistic 49.32. *P* < .0001. Log‐likelihood ratio (G squared) statistic 208.7, *P* < .0001.				

Model 2: Horses with Atrial fibrillation (AF, N = 164), are compared to horses performing to expectation (TE, N = 314).				
Variable	Median (IQR)	OR	OR 95% CI	*P* value
Intercept		0.06	0.01‐0.21	<.0001
Jurisdiction (HK vs AUS)		1.85	0.93‐3.84	.09
Distance behind winner (lengths)				
AF	20.93 (13.56‐29.88)	1.36	1.29‐1.45	<.0001
TE	4.28 (2‐6.75)			
Cumulative prizemoney (US $1000)/start				
AF	5.6 (1.8‐10.7)	1.02	1.01‐1.03	.006
TE	5.3 (1.8‐10.4)			
Age				
AF	4.8 (4.1‐6.1)	0.72	0.55‐0.92	.01
TE	5.0 (4.1‐6.0)			
Comparison with TE. Tjur's r^2^ = 0.64, area under ROC curve (95% CI) = 0.94 (0.92‐0.97), *P* < .0001. Hosmer‐Lemeshow statistic 18.97, *P* = .01. Log‐likelihood ratio (G squared) statistic 326.9, *P* < .0001.				

*Note*: Odds ratios reflect AF as the positive outcome.

Abbreviations: CI, confidence interval; EIPH, exercise induced pulmonary hemorrhage; IQR, interquartile range; kg, kilograms; km, kilometers; OR, log odds ratio; US $, US dollars.

#### 3.2.3 Analysis of postrace endoscopic findings

Postrace endoscopic examination was performed on 109/164 horses with AF and on 213/321 PP horses. Table 2 shows the number of horses in each group with EIPH and TMA grades 0‐4 and results of comparison. Horses with AF were at increased risk of having EIPH of any grade and of having clinically important EIPH.

**TABLE 2:** Number of horses in the atrial fibrillation (AF) and poor performance (PP) with exercise induced pulmonary hemorrhage (EIPH) and tracheal mucus accumulation (TMA) grades 0‐4.

	EIPH		TMA	
	AF N = 109	PP N = 213	AF N = 109	PP N = 213
	N (%)	N (%)	N (%)	N (%)
Grade 0	78 (71.6%)	187 (87.8%)	97 (89.0%)	173 (81.2%)
Grade 1	3 (2.8%)	10 (4.7%)	7 (6.4%)	17 (8.0%)
Grade 2	5 (4.6%)	9 (4.2%)	2 (1.8%)	19 (8.9%)
Grade 3	6 (5.5%)	5 (2.3%)	3 (2.8%)	4 (1.9%)
Grade 4	17 (15.6%)	2 (0.9%)	0 (0%)	0 (0%)

			Odds ratio (95% CI)	Fishers exact test *P* value
EIPH grade ≥1	31 (28.4%)	27 (12.2%)	2.9 (1.6‐5.2)	.001
EIPH grade ≥3	23 (21.1%)	7 (3.3%)	7.9 (3.3‐20.2)	<.0001
TM grade ≥1	12 (11.0%)	40 (18.8%)	0.54 (0.28‐1.0)	.08
TM grade ≥2	5 (4.6%)	23 (10.8%)	0.40 (0.16‐1.1)	.09

*Note*: Horses with AF were more likely to have EIPH of any grade, and more likely to have clinically important EIPH (≥3).

## 4 DISCUSSION

We identified several novel findings regarding the risk factors and consequences of postrace AF in thoroughbred racehorses. First, AF substantially impacted performance and we were able to quantify this impact under racing conditions, with horses with AF being distanced much farther behind the winner than other runners. However, lifetime earnings before diagnosis of AF was not impaired in this cohort. Second, exercise volume, as measured by age, number of starts, weight carried or race distances, was not found to promote AF. Finally, horses with AF were more likely to have clinically important EIPH than other poorly‐performing horses.

Atrial fibrillation impairs performance by decreasing cardiac output.^43^ The irregular heart rhythm results in fluctuations in ventricular filling, and stroke volume is further compromised by a lack of atrial contraction at end diastole.^43^ Previous studies have shown that AF is associated with poor racing performance.^2^, ^3^, ^5^, ^6^ The impact of AF on performance has been quantified under experimental conditions, with affected horses having a 12% decrease in velocity when in AF compared to when in sinus rhythm.^1^ In our study, the performance consequences of AF are detailed. Horses with AF finished approximately 21 lengths behind the winner. A study in standardbred racehorses, in which every competing horse was examined before and after the race, found that postrace AF occurred most often in horses that performed poorly.^2^ In that same study, other cardiac arrhythmias, including supraventricular premature depolarizations, ventricular premature depolarizations and ventricular tachycardia were not associated with poor performance.^2^ Atrial fibrillation occasionally is detected in horses that perform well, including winners, possibly because the onset of the arrhythmia occurred after the race finish.^2^, ^5^, ^43^ However, based on our findings, in horses that are distanced well back in the field, AF should be strongly suspected as a cause of poor performance.

Exercise‐induced pulmonary hemorrhage is highly prevalent in thoroughbred racehorses, being observed in 13% to 75% of tracheobronchoscopic examinations.^37^, ^44^, ^45^, ^46^, ^47^, ^48^, ^49^ Lower grades of EIPH have little to no impact on performance.^44^, ^49^ Severe EIPH (grades 3 and 4) is observed in fewer than 10% of examinations and can substantially impair performance.^44^, ^49^ We applied a previously established EIPH grading score^33^ and found that horses with AF were more likely to have EIPH of any grade, and more likely to have severe grades (≥3) of EIPH than other poorly‐performing horses. Horses in our study raced only in jurisdictions in which race day furosemide administration is prohibited. Compared to previous reports, horses in our study had a lower overall incidence of postrace EIPH, but horses with AF had a higher incidence of severe EIPH.^44^, ^45^, ^46^, ^47^, ^48^ The low overall incidence of EIPH might be related to the relatively short duration between race finish and endoscopic examination because examinations performed within 30 minutes of racing are likely to underestimate severity.^46^ The timing to examination in our study was dictated by the race day schedule. Although this might have been too soon to optimally examine the airways for EIPH, a longer time to examination could compromise the detection of AF, in cases in which it is short‐lived. Despite these limitations, a high incidence of severe EIPH was observed in horses with AF. An association between AF and EIPH has long been suspected, and epistaxis has been reported as a common presenting complaint in horses with AF.^27^ A previous study of thoroughbreds did not find an association between epistaxis and AF on race day.^3^ However, epistaxis is infrequent, and this association would be difficult to establish because of low study power. Exercise‐induced pulmonary hemorrhage is thought to arise as a result of stress failure of pulmonary capillaries associated with high intravascular pressure and negative pleural pressure.^38^, ^50^ High pulmonary arterial, pulmonary artery wedge and pulmonary capillary transmural pressures are observed in strenuously exercising horses.^51^ Left atrial pressure is a principal determinant of pulmonary capillary pressure, and AF is both a cause and consequence of increased left atrial pressure.^52^ During exercise, splenic contraction causes a rapid expansion of blood volume and increases left atrial and subsequently transmural pressures, which can precipitate EIPH.^52^ Atrial fibrillation impairs atrial distensibility and acutely induces further increases in left atrial pressure.^53^ This mechanism could explain the high incidence of severe EIPH observed in exercising horses with AF in our study.

Tracheal mucus accumulation is corelated with bronchoalveolar lavage cytology reflecting lower airway inflammation.^35^, ^54^ In racehorses, TMA most often is caused by mild to moderate asthma, with a variable contribution of viral and bacterial infection.^55^, ^56^, ^57^, ^58^ Asthma impairs gas exchange,^59^ and TMA grades ≥2 previously have been associated with impaired performance in thoroughbred racehorses and are considered clinically important.^36^ Tracheal mucus accumulation of any grade ≥1 was observed in 35% of thoroughbreds in the United Kingdom^56^ and 59% of thoroughbreds in the United States.^36^ The prevalence of TMA appears variable in Australia, with 1 recent study reporting 2.5%^37^ and another 45%^58^ of horses having TMA ≥1. Postrace endoscopic examinations identified a high prevalence of >99% in thoroughbred horses in South Africa^60^ and in barrel racing horses.^38^ Our study found similar prevalence of TMA compared with previous Australian studies,^37^, ^58^ but prevalence was lower than previous studies conducted postrace elsewhere.^38^, ^60^ In our study, a relatively low prevalence of clinically important TMA was found in AF horses. Higher grades of EIPH in this group might have obscured TMA during endoscopy. The low prevalence of TMA is in agreement with a prior study of horses exercised on a treadmill that found no association between the prevalence of arrhythmias and lower airway disease.^31^

It has been observed previously that most AF episodes detected in racehorses are paroxysmal.^3^, ^5^ Horses in our study were not definitively classified as having paroxysmal or persistent AF because they were not consistently examined before racing or in the days after. Horses susceptible to PAF can perform well between episodes,^5^ but are thought to exhibit erratic performance. There are inherent difficulties in comparing performance outcomes in studies of Thoroughbred racehorses.^44^ In our study, the cumulative prize money per start was marginally higher in AF horses compared to other groups in the multivariable model. This finding reflects that the overall career performance of AF horses is not impaired, and is in agreement with a prior study that found AF horses could perform well between episodes.^5^ We found that AF horses had decreased performance in the 0‐30 days before and including the event compared to control horses, but did not experience decreased performance at any other time interval. Short‐lived undiagnosed PAF episodes could impair other performances in addition to the diagnosed event, but we did not find strong evidence for this possibility at any interval in the preceding 180 days. Continuous monitoring devices and increased surveillance are needed to better understand the underlying burden and progression of PAF in racehorses.^61^

An association between volume of exercise training and risk for AF was not clearly established in our study. We found limited evidence in the univariable analysis for AF horses having a higher average race distance compared to other poor performers. We also found a modest decrease in the frequency of racing for AF horses in the 30 days before the event. Horses undergo cardiac structural, functional and electrical remodeling in response to race training.^23^, ^24^, ^25^, ^26^ This remodeling enhances athletic performance through increased stroke volume.^62^ In human athletes, exercise‐induced cardiac remodeling promotes cardiac arrhythmias, most importantly, AF and atrial flutter.^62^, ^63^ Training‐induced electrical remodeling has been observed in standardbred racehorses as evidenced by a decrease in resting heart rate, increased prevalence of second‐degree atrioventricular block, changes in ventricular repolarization and prolongation of the PR interval.^25^, ^26^ Other variables that would be expected to reflect volume of exercise training, including age and cumulative race distance, were not associated with AF in our study. Age was retained in both multivariable models, with AF horses being marginally younger than PP or TE horses. This observation is in contrast to previous studies in thoroughbreds^3^ and standardbreds^2^ that found an association between increasing age and AF development. Increasing age, increasing career starts and higher lifetime race distance have been emphasized as a risk factors for sudden death in racehorses.^64^, ^65^ However, another study investigating sudden cardiac death specifically found younger horses, with fewer career starts, to be at highest risk.^66^ The jurisdictions in which our study was conducted (Victoria, Australia, and HK) both have restrictions to ongoing participation of horses with a history of postrace AF. In these jurisdictions, horses are subject to mandatory stand downs and veterinary clearance examinations. A previous study in HK found approximately 25% of horses were retired immediately after diagnosis of their first AF episode.^5^ Increased detection of AF through screening programs implemented by the jurisdictions involved in this study might have fostered early retirement of these horses. We suspect that these practices explain the younger age of AF horses in this cohort, because horses vulnerable to AF likely were removed from the population through retirement. In our study, only records relating to race distance were accessed. Exercise distance and speed undertaken during training vary widely.^67^ Differences in training volume on the risk of AF could not be evaluated in our study but would be worthy of consideration in future studies.

The observation of decreased frequency of racing immediately before the event was a surprising finding. Decreased frequency of racing has been associated with increased risk for sudden death^64^ and catastrophic musculoskeletal injury in horses.^68^, ^69^ Undertraining and overtraining before competition both increase the risk of injury in human athletes.^70^ Training improves cardiovascular efficiency and results in a decreased heart rate for a standard workload.^71^ Maximal heart rate reflects exercise intensity and is associated with the frequency of arrhythmias in exercising horses.^72^ In addition to exercise intensity, heart rate also is affected by autonomic tone and familiarity with a task.^73^ Changes in autonomic tone associated with unfamiliar exercise workloads can increase the prevalence of arrhythmias in horses.^74^ The observation of decreased racing frequency before the detection of AF in horses in our study might reflect inadequate fitness. Horses with decreased racing frequency and consequent lower exposure to racing also might be more excitable, which would be expected to cause fluctuations in autonomic tone. Both inadequate fitness and higher fluctuations in autonomic tone have the capacity to affect arrhythmia vulnerability.

A limitation of our study centers on the detection of PAF in horses. In our study, horses that performed below expectation on race‐day were identified for veterinary inspection by stipendiary stewards. Therefore, horses that had better historical performance before the event might have been more likely to be selected for inspection. This situation might explain the finding of better performance in PP horses in the 30 to 60 days before to the event. We attempted to account for this potential source of bias by including both horses that performed poorly and were selected for inspection, as well as horses that performed to expectation, for comparison to AF horses. Only the horses performing below expectation (both AF and PP) were examined by a veterinarian postrace. Other horses in the field might have had episodes of AF that were not diagnosed. Furthermore, in those that were examined, PAF episodes might have converted to sinus rhythm before examination. Therefore, the potential for misclassification of horses existed. In addition, horses performing to expectation did not have respiratory endoscopy, which precluded a comparison of EIPH and TMA in AF horses and horses performing to expectation. Our study involved only 2 racing jurisdictions, and these findings might not be applicable to other regions. Hong Kong also has a predominantly male racehorse population, and therefore any association between AF and sex could not be assessed. In other studies, no difference in the prevalence of AF based on sex has been identified.^3^, ^30^

In conclusion, we found that acute AF episodes substantially impaired race performance in thoroughbred horses, but career earnings were not inferior. Atrial fibrillation should be suspected as a strong possibility for poor performance in horses distanced well behind the winner. We did not find evidence for an influence of exercise volume on AF development in our study. Lastly, horses with AF had increased frequency and severity of EIPH. These findings suggest a mechanistic relationship between AF and EIPH in strenuously exercising horses.

## CONFLICT OF INTEREST DECLARATION

Authors declare no conflict of interest.

## OFF‐LABEL ANTIMICROBIAL DECLARATION

Authors declare no off‐label use of antimicrobials.

## INSTITUTIONAL ANIMAL CARE AND USE COMMITTEE (IACUC) OR OTHER APPROVAL DECLARATION

Approved by the University of Adelaide Animal Ethics Clearance, S‐2017‐088.

## HUMAN ETHICS APPROVAL DECLARATION

Authors declare human ethics approval was not needed for this study.

## Supporting information


**Supplementary Item S1.** Definition of below expectation performance.

**Supplementary Table S1.** Findings from Kruskal‐Wallis test with Dunn's correction for multiple comparisons investigating horses with AF and poorly performing horses and horses performing to expectation (AF = Atrial Fibrillation. PP = Poorly performing group. TE = To expectation group. IQR = Interquartile range. N = number of horses. OR = log odds ratio. CI = confidence interval. m = meters. Kg = kilograms. $US = US dollars).

**Supplementary Table S2.** Findings from univariable logistic regression between horses with postrace atrial fibrillation (AF, N = 164) and poorly performing horses (PP, N = 321) and horses performing to expectation (TE, N = 314) evaluating race day, historical data, and respiratory data. (IQR = Interquartile range. N = number of horses. OR = log odds ratio. CI = confidence interval. km = kilometers. Kg = kilograms. $US = US dollars. EIPH = exercise‐induced pulmonary hemorrhage).
