# Supplementary material for: Associations between postrace atrial fibrillation and measures of performance, racing history and airway disease in horses
Source: J Vet Intern Med. 2023 Sep 23;37(6):2573–83. doi: 10.1111/jvim.16878 (PMC10658555; doi:10.1111/jvim.16878)
Supplement: Supplementary file 1 — Supplementary Item S1. Definition of below expectation performance. [file JVIM-37-2573-s002.pdf]

Definition of below expectation performance and determinants of post-race inspection.

The present study was designed to utilise veterinary records retrospectively obtained which identified horses with post-race atrial fibrillation. Horses were identified with atrial fibrillation following post-race inspection by the attending veterinarian. These inspections take place at the discretion of raceday stipendiary stewards. Horses are selected for veterinary inspection by the stewards based on a below expectation performance. The determination of a 'below expectation' performance is made following evaluation of the predicted placing of the horse within the field. The predicted placing is largely based on previous performance and pre-race betting odds. In our study, atrial fibrillation cases were identified from this population of horses inspected post-race. To account for the possibility of bias, we chose another group of horses that had also been selected for post-race veterinary inspection. The second control group, those performing 'to expectation' were not subject to any veterinary inspection and were considered to represent a group of normally performing horses.

The following is taken from:

Racing Victoria. *Raceday responsibilities*. 2022; Available from: https://www.racingvictoria.com.au/the-horse/veterinary-care/raceday-responsibilities

"Post Race Inspections

Veterinary surgeons inspect all horses as they leave the racetrack for signs of injury, distress or conditions that may occur as a result of strenuous physical exercise. The stipendiary stewards may request that a comprehensive clinical examiination be performed on horses that perform below expectations, were involved in racing incidents or were observed to show signs of injury or other physical abnormalities.

It is important to identify any veterinary reasons for any abnormalities that may have been observed in running or return from racing for the following reasons:

- Disappointing performances caused by veterinary problems may be explained to the public.

- Any injuries or infirmities can be identified and referred for appropriate treatment.
- To ensure that any horse-related factors can be taken into account during an inquiry into the running of a horse.
- To assist handicappers in assessing the relative merit of a horse's performance on the day of racing.

Sometimes it is not possible to detect a condition that may have affected racing performance immediately after the race. In some cases lameness or illness may only become apparent on the day after racing. For this reason Australian rule of racing AR 140 requires that trainers must report any condition detected in a horse under their care that may have affected a horse's racing performance.

Assessing Poor Racing Performance

While trainers and their veterinary surgeons work hard to present their horses to race in the best possible physical and mental condition, occasionally a horse will suffer a physical condition that will cause it to return a racing performance below that which was expected of it on the day.

Conditions that may affect racing performance include lameness, laceration, EIPH, heat stress, breathing abnormalities including laryngeal hemiplegia, dorsal displacement of the soft palate and epiglottic entrapment, and atrial fibrillation, eye injuries and others.
