# Supplementary material for: Associations between postrace atrial fibrillation and measures of performance, racing history and airway disease in horses
Source: J Vet Intern Med. 2023 Sep 23;37(6):2573–83. doi: 10.1111/jvim.16878 (PMC10658555; doi:10.1111/jvim.16878)
Supplement: Supplementary file 2 — Supplementary Table S1. Findings from Kruskal‐Wallis test with Dunn's correction for multiple comparisons investigating horses with AF and poorly performing horses and horses performing to expectation (AF = Atrial Fibrillation. PP = Poorly performing group. TE = To expectation group. IQR = Interquartile range. N = number of horses. OR = log odds ratio. CI = confidence interval. m = meters. Kg = kilograms. $US = US dollars). [file JVIM-37-2573-s003.pdf]

Supplementary Table 1. Findings from Kruskal-Wallis test with Dunn's correction for multiple comparisons investigating horses with atrial fibrillation and poorly performing horses and horses performing to expectation. (AF= Atrial Fibrillation. PP= Poorly performing group. TE = To expectation group. IQR = Interquartile range. N = number of horses. m = metres. Kg = kilograms. $ US = US dollars.)

Variable Median (IQR)	N = 164 AF horses	N = 321 PP horses	N = 314 TE horses	Kruskal-Wallis P value	AF vs PP P value	AF vs TE P value	PP vs TE P value
**RACE DAY**							
Rating	62 (53-75)	64 (52-77.5)	62.5 (52-75)	0.77	>0.99	>0.99	>0.99
Race distance (m)	1400 (1200-1650)	1400 (1200-1650)	1400 (1200-1650)	0.78	>0.99	>0.99	>0.99
Weight carried (kg)	57 (55-58.5)	57 (55-59)	57 (55-58)	0.29	>0.99	>0.99	0.36
Distance behind winner (lengths)	20.93 (13.56-29.88)	7 (4.8-9.8)	4.3 (2-6.8)	<0.0001	<0.0001	<0.0001	<0.0001
**HISTORICAL DATA**							
Age (years)	4.8 (4.1-6.2)	5.0 (4.1-6.0)	5.0 (4.1-6.0)	0.84	>0.99	>0.99	>0.99
Cumulative starts	14 (7-21)	13 (7-23)	14 (6-26)	0.76	>0.99	>0.99	>0.99
Cumulative distance (m)	21600 (9850-37663)	17963 (8350-35983)	20245 (8200-38250)	0.54	0.84	>0.99	>0.99
Cumulative prizemoney ($ US)	77219 (20306-215342)	72006 (18916-201378)	72611 (15678-196833)	0.59	>0.99	>0.99	>0.99
Cumulative distance/ start	1418 (1202- 1685)	1356 (1187- 1574)	1365 (1200-1590)	0.05	0.04	0.20	>0.99
Cumulative ($ US) / start	5587 (1881-10689)	5299 (2439- 11339)	5271 (1745- 10427)	0.49	>0.99	>0.99	0.79
**INTERVAL DATA**							
Cumulative starts, days prior							
0-30	2 (1-2)	2 (1-2)	2 (1-2)	0.005	0.03	0.004	>0.99
30-60	1 (0-1)	1 (0-2)	1 (0-1)	0.07	0.06	0.51	0.80
60-90	1 (0-1)	1 (0-1)	0 (0-1)	0.98	>0.99	>0.99	>0.99
90-180	2 (0-4)	2 (0-3)	2 (0-3)	0.25	0.63	0.30	>0.99
Distance (m), days prior							
0-30	2400 (1400-3475)	2600 (1650-3450)	2600 (1650-3400)	0.61	>0.99	0.98	>0.99
30-60	1200 (0-2200)	1200 (0-2400)	1200 (0-2200)	0.39	>0.99	>0.99	0.55
60-90	1200 (0-1650)	1000 (0-1650)	0 (0-1650)	0.54	>0.99	0.95	>0.99
90-180	2400 (0-5000)	2200 (0-4800)	2400 (0-4200)	0.48	>0.99	0.68	>0.99
Prizemoney ($ US), days prior							
0-30	0 (0-5371)	1428 (0-9439)	2831 (0-14666)	<0.0001	0.007	<0.0001	0.12
30-60	0 (0-4784)	455 (0-10877)	0 (0-4459)	0.001	0.01	>0.99	0.003
60-90	0 (0-896)	0 (0-4220)	0 (0-1129)	0.26	0.56	>0.99	0.45
90-180	2695 (0-34164)	1034 (0-20102)	750 (0-12625)	0.19	0.69	0.20	>0.99
Distance/ start, days prior							
0-30	1400 (1200- 1800)	1400 (1200-1700)	1400 (1200-1650)	0.32	0.57	0.47	>0.99
30-60	1400 (1200-1750)	1400 (1200- 1650)	1400 (1200-1600)	0.22	0.98	0.25	>0.99
60-90	1400 (1200-1650)	1400 (1200- 1650)	1400 (1200- 1650)	0.23	0.35	0.27	>0.99
90-180	1380 (1200-1600)	1400 (1200- 1550)	1350 (1200- 1600)	0.89	>0.99	>0.99	>0.99
Prizemoney ($ US) / start, days prior							
0-30	0 (0-2324)	703 (0-4720)	1540 (0-7955)	<0.0001	0.008	<0.0001	0.04
30-60	1616 (0-9483)	4345 (481- 15570)	1709 (0-7261)	0.002	0.09	>0.99	0.002
60-90	435 (0-7643)	3478 (0-10701)	1470 (0-9441)	0.21	0.28	>0.99	0.75
90-180	5127 (457- 11916)	3678 (336 -10580)	2675 (223-8875)	0.23	>0.99	0.27	0.97
