# Supplementary material for: Associations between postrace atrial fibrillation and measures of performance, racing history and airway disease in horses
Source: J Vet Intern Med. 2023 Sep 23;37(6):2573–83. doi: 10.1111/jvim.16878 (PMC10658555; doi:10.1111/jvim.16878)
Supplement: Supplementary file 3 — Supplementary Table S2. Findings from univariable logistic regression between horses with postrace atrial fibrillation (AF, N = 164) and poorly performing horses (PP, N = 321) and horses performing to expectation (TE, N = 314) evaluating race day, historical data, and respiratory data. (IQR = Interquartile range. N = number of horses. OR = log odds ratio. CI = confidence interval. km = kilometers. Kg = kilograms. $US = US dollars. EIPH = exercise‐induced pulmonary hemorrhage). [file JVIM-37-2573-s001.pdf]

Supplementary Table 2. Findings from univariable logistic regression between horses with post-race atrial fibrillation (AF, N=164) and poorly performing horses (PP, N=321) and horses performing to expectation (TE, N=314) evaluating race day, historical data and respiratory data. Comparisons between AF and PP horses and AF and TE horses were performed separately and have been merged in the table for brevity. Odds ratios reflect AF as the positive outcome. (IQR = Interquartile range. N = number of horses. OR = log odds ratio. CI = confidence interval. km = kilometers. Kg = kilograms. $ US = US dollars. EIPH = exercise induced pulmonary hemorrhage)

Variable	Median (IQR)	OR	95% OR	Likelihood ratio test P value
**RACE DAY**				
Rating				
AF	62 (53-75)			
PP	64 (52-77.5)	1.0	0.99 to 1.01	0.96
TE	62.5 (52-75)	1.0	0.99 to 1.01	0.55
Race distance (km)				
AF	1.4 (1.2-1.7)			
PP	1.4 (1.2-1.7)	1.0	0.60 to 1.63	0.99
TE	1.4 (1.2-1.7)	1.1	0.65 to 1.87	0.69
Weight carried (kg)				
AF	57 (55-59)			
PP	57 (55-59)	1.00	0.99 to 1.03	0.99
TE	57 (54.5-58)	1.01	0.98 to 1.05	0.60
Distance behind winner (lengths)				
AF	20.93 (13.56-29.88)			
PP	7.00 (4.75-9.75)	1.17	1.13 to 1.21	<0.0001
TE	4.28 (2.00-6.75)	1.32	1.26 to 1.40	<0.0001
**HISTORICAL DATA**				
Age				
AF	4.8 (4.1-6.1)			
PP	5.0 (4.1-6.0)	1.02	0.89 to 1.17	0.78
TE	5.0 (4.1-6.0)	0.97	0.85 to 1.11	0.67
Cumulative starts				
AF	14 (7-21)			
PP	13 (7-23)	1.00	0.99 to 1.02	0.65
TE	14 (6-26)	1.00	0.98 to 1.01	0.68
Cumulative distance (km)				
AF	21.6 (9.9-37.7)			
PP	18.0 (8.4-36.0)	1.00	0.99 to 1.01	0.46
TE	20.2 (8.2-38.3)	1.00	0.99 to 1.01	0.96
Cumulative prizemoney ($1000 US)				
AF	77.2 (20.3-215.3)			
PP	72.0 (18.9-201.4)	1.00	1.00 to 1.00	0.06
TE	72.6 (15.7-196.8)	1.00	1.00 to 1.00	0.13
Cumulative distance (km)/ start				
AF	1.4 (1.2- 1.7)			
PP	1.4 (1.2- 1.6)	1.55	1.05 to 2.48	0.02
TE	1.4 (1.2- 1.6)	1.43	0.99 to 2.30	0.06
Cumulative prizemoney ($1000 US) / start				
AF	5.6 (1.8-10.7)			
PP	5.3 (2.4- 11.4)	1.01	1.00 to 1.02	0.048
TE	5.3 (1.7- 10.4)	1.01	0.99 to 1.02	0.14
